# Pelvic Abscess*Read at meeting of East Texas Medico-Chirurgical Society, January 10, 1900.

**Published:** 1901-06

**Authors:** R. M. Dunn

**Affiliations:** Palestine, Texas


### TEXAS MEDICAL JOURNAL.

ESTABLISHED JULY, 1885.

PUBLISHED MONTHLY.—SUBSCRIPTION $1.00 A YEAR.

VOL. XVI. AUSTIN, JUNE, 1901. No. 12.

### Original Contributions.

For Texas Medical Journal.

### Pelvic Abscess.*

R. M. DUNN, M. D., PALESTINE, TEXAS.

DEFINITION.

By pelvic abscess we mean a circumscribed, or non-circumscribed collection of pus within the pelvic cavity. Characterized by pain of a peculiar throbbing, boring nature, constant fever (99½ to 104), a more or less symmetrical elastic indurated swelling felt per vaginam.

ETIOLOGY.

This subject is so closely related to pelvic inflammation, of which it is one of the results, that we must ask you to bear in mind the relation they sustain to each other, and if it seems that my subject is somewhat disconnected in causation and symptoms, it will only be apparent, and not real, for the same thing that causes a pelvic inflammation may, and does, cause pelvic abscess.

We recognize two main causes for these abscesses, "Septic and Specific"; each gaining entrance to the peritoneum, and cellular tissue by various modes and routes.

SEPTIC.

These are the cases in which, if of recent origin, you will find one

*Read at meeting of East Texas Medico-Chirurgical Society, January 10, 1900.

form of pus producing cocci; mainly the streptococcus and staphylococcus. They usually find entrance to the pelvic cavity through some form of traumatism; as child birth, badly managed abortions, lacerations of the cervix uteri and pelvic floor. Traveling thence through the lymph channels, to the pelvic cellular tissue. Where there are no solutions of continuity, the infection is usually introduced into the vagina and uterus by dirty instruments, and intra uterine applications, including dilations, curettings, operations through the abdominal walls, on the ovaries, tubes, and uterus. I remember in this connection a case I operated on, removing the ovaries and stitching the uterus to the abdominal wall. The patient did nicely for two or three days; suddenly her temperature went up to 103½ to 104½. On the fifth day she had a rigor. The abdomen was not tender or swollen except just above the pubes. Defecation gave great pain, a round elastic tumor filled Douglass' pouch. I opened this on the tenth day, after operation. It was filled with pus. The temperature dropped to normal within the next twenty-four hours, and did not again rise. The cavity rapidly contracted and healed, the patient making from that time on an uneventful recovery. This infection may also reach the peritoneum through the vagina to the pelvic cavity by continuity along the mucous membrane of the vagina, uterus and tubes.

SPECIFIC.

Gonorrhea, in my opinion, is responsible for more cases of pelvic suppuration than any other one element. It is not, however, more frequently found. Many abscesses opened in the pelvis contain no pus producing organisms at all.

These, I think, were primarily gonorrheal; at least the most of them, and in their natural evolution have disappeared. These abscesses would in time become reinfected with bacteria of another kind.

PATHOLOGY.

The abscess from whatever cause, or part involved, is the same, and is only a matter of anatomical limitation, the extent depending on the nature of infection, resistance of the tissue involved, and the ability of nature to quickly throw out its protecting lymph to wall it off, should it be in the pelvic cavity. If, on the other hand, it has spread through the lymph channels, and the cellular tissue is primarily involved, then the same zone of demarkation as seen in phlegmons and gangrene in other situations must be looked to for its limitation.

Infection entering the tube sets up at once either an acute or sub-acute local reactionary inflammation, sealing in a very short time the fimbriated and uterine ends of the tube. The suppurative process, if involving only one end, or the whole tube, may burst through the uterine end, or slowly make its way by suppurative degeneration, emptying its contents into the cavity of the uterus.

If carefully looked after at this point the tube may regain its normal anatomical and physiological appearance and function. Should the fimbriated end slowly give way, there will be found a tube-ovarian abscess, involving the fimbriated end of the tube and the ovary; even the body of the ovary may be involved. The pus may travel along the tube and open into the uterus. This was the condition observed in a case I did a hysterectomy on for fibroids. The abscess, however, was not suspected; the ovarian enlargement being the size of an orange with thick indurated walls, was taken for a nodule of the tumor. The tube coats were much thickened and sclerosed with a well formed pyogenic membrane lining the tube and abscess cavity. This condition had no tendency to a spontaneous cure.

If neither end of the tube opens, the entire tube may form one large abscess, gradually increasing in size, the walls becoming thinner, the outer peritoneal coat inflamed and adherent to surrounding structures. Several different pockets may form having no connection whatever with each other. I remember operating on a case that had four distinct sacs in the same tube. The tube when distended is one single cavity, or many different sacs becomes much elongated and coils, giving the appearance of a conch.

The walls of the tube may not stretch, but by infiltration, exudation and the invasion by the cocci, become much thickened and friable; the vessels only resisting the cutting through of a ligature when applied. In this condition the cocci may penetrate the tube wall and invade the pelvic peritoneum. As soon as the pus reaches the peritoneal surface, or approaches near enough to cause a reactionary inflammation, plastic material is thrown out around the offending cocci and walls them off. If the cocci invade this mass of protecting lymph it is sure to suppurate; and the abscess cavity will nearly always be found occupying the space between the rectum and broad ligament of the side affected. If the bacteria do not reach this protecting lymph, only a hard induration can be felt, which, however, will only be found to occupy the peritoneum. When the infection enters through the lymph channels, the pelvic cellular tissue is primarily involved, extending ultimately to the peritoneum.

The entire pelvic cellular and peritoneal tissue being involved, an abundance of plastic lymph is thrown out, and the different organs are glued together in the position they happen to be at the time. There is an abundance of exudation into all the pelvic tissue, producing thickening and swelling, the whole roof of the vagina feels as hard as frozen meat. The uterus is immovable and gives to the examining finger the impression that it was moulded in that position.

The position of none of the pelvic organs can be located. If the suppurating cocci reach this inflammatory deposit, it may break down in one or several places. Only a small focus may suppurate, or the whole pelvic cavity may be filled with pus, the uterus being in the center, posterior, or to one or the other sides. It may occupy

Douglass' pouch and one side; then the ovary and tube will be above the abscess. In cases of such extensive suppuration the uterus and its appendages, when the cavity is emptied, contracts and heals, will be bound by adhesions in some abnormal position. These adhesions are rarely absorbed and the uterus will thus remain indefinitely in this position.

### CLINICAL HISTORY.

The clinical history will vary according to the cause, parts invaded, and amount of tissue included in the suppurative process.

If it be a streptococcus infection, you will usually get a history of an abortion, labor at term, intra uterine applications or injections. The gonorrheal case will give you an entirely different history. She has had an old "womb complaint"; if she is a married woman it will date from her first marriage week, or from an early abortion after which she has never again been well, or become pregnant. Coitus or any movement of the pelvic organs will be painful. This condition gradually grows worse, and finally, in an emaciated condition, she keeps her bed. Slight chills and a degree or so of fever may be present. In a few of the cases that suppurate early there will be no emaciation. In the streptococcus cases a rapid invasion with or without the history of the gonorrheal infection, will be noted. Exceptionally an acute attack of gonorrhea will rapidly go on to suppuration. The condition and appearance of a patient with a gonorrheal suppuration of the ovaries, tubes, pelvic peritoneum, and connective tissue, varies from that of extreme emaciation to that of perfect health.

I have examined patients in my office who were in a good state of general health, that had large adherent pus tubes and abscesses in different positions about the uterine appendages. These patients usually applied for relief for "falling of the womb," or profuse blood streaked leucorrhea and painful menstruation.

### SYMPTOMATOLOGY.

The symptoms will vary in accordance with the anatomical parts attacked, the intensity and cause of the primary inflammation.

We may, therefore, divide the causes into two main groups: acute and sub-acute. The acute form you will usually find coming on after normal labors, abortions, operations and intra-uterine applications.

These are the cases that we designate as septic; and you will usually find some one of the pus cocci in the abscesses.

The symptoms of these cases are tolerably uniform, beginning usually with a distinct chill or rigor, followed by fever 103 to 104, rapid pulse 120 to 130 full and bounding, hot skin, flushed countenance, headache, furred tongue, nausea, vomiting and restlessness. The abdomen is tender, especially the lower portion. Constipation is the rule, and when the bowels do act it causes great pain and tenesmus.

The urine is scanty, high colored and passed with much pain. The acute may, if not interfered with, gradually merge into the chronic form. The chronic or sub-acute form begins so insidiously that the abscess may be found accidentally while examining the woman for some other supposed trouble, or while doing some operation. Usually, however, you will have before you an unmarried woman, and one of easy virtue. She is not old, but began life early.

She will give you a distinct history of gonorrhea, since which time she has kept up some kind of treatment for leucorrhea.

If she is a married woman she will tell you that she had at some previous time a profuse, irritating leucorrhea, with dysurea and swollen genitals. The husband, if he will tell the truth, will acknowledge to the "running of the range" at the same time. Sometimes she will tell you that this leucorrhea come on the first two weeks of her marriage, after which she has not been well again.

Her menses are always painful and irregular. Walking causes a dragging sensation in the lower abdomen. Her appetite is bad, furred tongue, bowels constipated and their movements painful. These slight symptoms slowly increase, and after having had several attacks of slight pelvic inflammation which left her in a little worse condition than the one preceding, she commits some excess, such as too frequent coitus, a cold bath to stay the period, because it happened to be on hand when a busy night was expected, sudden suppression from cold or excitement, excessive walking or scrubbing; in fact, anything that disturbs the equilibrium of the pelvic tissues and organs.

This slight disturbance, from whatever cause, will be sufficient to keep her in bed a part or all the time with irregular fever and weak rapid pulse. There may be a distinct chill, but more often it is only chilly sensations that are complained of. The head aches and feels heavy. The tongue gradually takes on a thick, dirty, brown coat. The appetite is lost and nausea and vomiting become a prominent symptom. So prominent is this symptom in some cases that your patient will appeal to you for its relief, stating that her excessive vomiting is the cause of the pains and soreness in her abdomen.

I remember seeing two cases in which this was so prominent, that they had been for nearly two months treated for gastritis. One of these had a large abscess on the right side, and the other the entire pelvic cavity was filled with pus. There is now constant pain in the abdomen, increased by pressure or movement. It is of a throbbing nature and usually more severe at night. The face has a peculiar drawn appearance, which betokens much suffering. With loss of sleep, appetite and the deleterious action of the septic material on the cells of the body, emaciation is marked and in some cases extreme.

I remember having seen a case that the history indicated that she had an abscess discharging into the rectum at four or five months intervals for five or six years. She was greatly emaciated, suffering all the ills that a hectic-septic patient is heir to. She had a slight cough and night sweats, and was supposed by her friends and relatives to be dying of consumption. No amount of tonics or forced feeding would improve her condition. After removing the abscess, however, she rapidly regained her former health and flesh. This has been five years and she still remains perfectly well, with little or no pain at her menstrual periods, which are regular and normal. Usually the periods are irregular, and may appear every two weeks. In a certain number of cases a necrotic, thick, bad smelling, bloody discharge will escape from the uterus. The abdomen may or may not be swollen; deep pressure on one or the other side will elicit pain.

With these facts before you I think you will appreciate the necessity for a digital examination, upon which depends your diagnosis. I appeal to you to make this examination with the utmost care and precision, for upon this alone depends your intelligent management of the case.

The patient should be on a good hard mattress in the dorsal position, with knees drawn up, and all clothing removed, except a loose gown. You will now seat yourself by the side of the bed facing the patient. You must arrange the bed clothing so that the hand can be carried between the legs, and not in the classic obstetrical position, between the leg and thigh next to you. If you use the latter position, it will be almost impossible to explore the opposite side of the pelvis without changing sides. With the index and middle fingers well oiled the vagina is carefully entered. This will be found in acute cases, hot with pulsating arteries. In the sub-acute about normal. You must first seek and recognize the cervix uteri, and note its position. If the abscess is on one side it will be high up and on the opposite side. If in Douglass' pouch, it will be high up and resting under the pubic arch. If on both sides it may be normal. Your next task is to locate the body of the uterus. With the index or middle finger on the cervix and the other hand on the abdomen, you will try to locate the body. You will seldom be able to do so, and but little time should be spent in this effort; the uterine sound introduced without force through a Hale's speculum is the only means by which the fundus can be located. You have noticed the position of the cervix and body of the uterus, their range of mobility and tenderness. You will now proceed to locate any tumor or abnormality in the vaginal roof. While you are making this examination watch carefully the expression of the patient's face. It will be an index to the pain your finger is producing, and will be of much service to you in locating the tender places. In the acute cases the roof will generally be hard, smooth and the appendages will be immovable. If the suppurative process has been going on for some time you will usually find one or more tender places in the board-like roof of the vagina. This with the history should be sufficient to lead to the next step, aspiration, if necessary.

In the chronic cases the vaginal roof is not generally so hard, and the uterus is nearly always to one side or anterior, and a distinct swelling, either boggy or fluctuating, will occupy its place. The tumor if outside of the tube will be rounded and smooth, if confined to the tube it will be elongated and crooked, giving the impression to the examining finger of a recently stuffed sausage, and a distinct sulcus can be felt between it and the uterus. When you have satisfied yourself as to the condition felt per vaginum, you should extend the investigation to the rectum. Here you can explore some distance above the cervix, and in most cases you will gain valuable information. In a majority of cases you will find a complete collar of deposit around the rectum giving the impression to the examining finger of passing through a semi-elastic ring. The tumor can be more distinctly made out, and fluctuation more evident than by vaginal examination. I have seldom been able to get true fluctuation, and am always satisfied if the tumor has a distinct elastic feel, indurated walls and septic symptoms. I have yet but one error to record. If the history and physical examination do not satisfy you, and a distinct elastic tumor can be felt, you should always aspirate. In a certain number of cases all the history, symptoms, and cause will point beyond doubt to a suppurative process in the pelvic cavity, yet the physical signs will be negative. In these cases you will not do an exploratory aspiration, but an exploratory operation through the abdominal wall.

### DIFFERENTIAL DIAGNOSIS.

Pelvic abscess will need to be differentiated from cyst. Fibroid tumors, ectopic gestation, hematocele, malignant deposits, and appendicitis. One or more of these may exist at the same time with the abscess. You will then, in addition to the history and symptoms of the abscess, have the history and symptoms of the additional trouble. A careful consideration of the history and septic symptoms will materially aid in the differentiation. In cyst and hydrosalpinx you will have an elastic or fluctuating tumor without indurated walls or septic history. Fibroid tumors will have a history of slow painless growth and profuse metrorrhage. They are hard and nodular to the touch.

From ectopic gestation you will need to consider the history of both. In ectopic gestation you will get a history of amenorrhea and suspected pregnancy, spasmodic pains, absence of fever, irregular hemorrhage and slow growth of the tumor.

Hematocele begins abruptly with evidence of internal hemorrhage. Occurs at or near menstral period. Little pain and no elevation of temperature. The tumor has a sensation as though it were filled with a semi-solid material, and its walls are not indurated.

In malignant deposits the cervix is usually involved, ulcerated and bleeding readily on any irritation, bad smelling seres or bloody discharge. The deposit is nodular, hard and usually surrounds the entire cervix. Pain worse at night than in the day.

In appendicitis you will have to depend on the symptoms, history and location. Usually the genital apparatus is not involved, and the pain is higher up. If, however, the appendix is low down, as it is in some cases, the two cannot be differentiated.

### PROGNOSIS AND TERMINATION.

The prognosis and termination will vary according to the cause, whether it be septic or specific, extent and resistance of tissue involved, condition and environment of the patient, and the time she comes under observation.

The prognosis in pelvic suppuration is good so far as life is concerned; but the uterine appendages in the majority of cases are seriously and permanently impaired. If the abscess is small and easy to reach per vaginam, or readily enucleated by the abdominal route, the prognosis is favorable. If there are large dense adhesions, or the cavity cannot be reached per vaginam, the prognosis is worse. If the fever is not high and the patient is in fairly good condition, the better the prognosis. Previous good health adds to the patient's chances of recovery. The prognosis is more serious if the abscess has found an opening by some tortuous route. Complications, such as fibroids, cyst, or malignant growths render the prognosis worse.

The suppuration may terminate when involving only the tube and discharging into the uterus in resolution. It may also terminate in resolution when a free spontaneous opening occurs into the vagina. Its tendency, if not interfered with, is to rupture or find an outlet somewhere. This may be into the uterus, vagina, rectum, bladder, abdominal wall, or into the peritoneal cavity. Its termination is usually fatal when it discharges into the peritoneal cavity. If the abscess discharges intermittently, or through long inaccessible sinuous tracks, the patient's powers will be worn out, or prove the exciting cause of some tubercular disease.

### TREATMENT.

The treatment of pelvic suppuration will vary according to the condition of the patient when first seen, the stage and location of the abscess. I have never been able to build up a patient who had pus in the pelvic cavity. It has been and is still my rule to operate as soon as possible. If the abscess has ruptured, and the patient is improving, I wait and give her a chance to recover without operation. Should her condition remain the same or change for the worse my plan is to operate. If the pus is walled off and the roof of the vagina is its anterior wall, I invariably open through the vagina. If it is a pyosalpinx and adherent to the roof of the vagina, I operate it in the same way. Sometimes it is impossible to say whether it is adherent or not. My rule is if it rest firmly on the vaginal roof, and has no free movement, evacuate per vaginam. If the pyosalpinx is movable or the tumor cannot be defined by the examining finger, and the vaginal roof is not hard, abdominal section is indicated. If the vaginal roof is hard and the patient has suppurative history, I first do an exploratory aspiration. If pus is reached I open through the vagina as usual. In preparing for this operation you must bear in mind that many complications and accidents may and do happen in these cases, even an abdominal section may become imperative. You should therefore make the same preparation that you would for an abdominal section. Profit by my experience and never try to do this operation with the woman across the bed. You will need a good table and three assistants. If your table has leg holders you can manage with two. The patient's kidneys and bowels should be looked after two or three days prior to that set for the operation. The day of operation she should have an enema and bath, a competent nurse, or yourself, should wash, scrub and shave the pubis and abdomen and cover with an aseptic towel, some hours before the time set for the operation. The following instruments should be sterilized and taken: long curved trocar, long curved sharp pointed scissors, (scissors for other work), pair uterine dilators, uterine sound, catheter, tenaculæ, curved needles, needle holder, dressing forceps, aneurism needle, half dozen artery forceps, aspirator, irrigator, Sim's speculum, peritoneal retractor, retractors, bistory and suitable ligatures, dressing, etc.

The patient can be given the anesthetic either in bed or on the table. The patient is now on the table in the dorsal position, with a Kelly's pad in place and the knees drawn up. You should not forget to draw her urine, after which you will cleanse the vagina with soap, brush and fountain syringe. You will now take advantage of the relaxation from the anesthetic to make a more careful examination than you have heretofore been able to do, and decide more fully where you will make your puncture. Before proceeding to operate you will again cleanse the vagina and your hands. You should now with the uterine sound locate carefully the fundus of the uterus. This should be your last duty before operating, as it may change its position and give serious trouble, as happened to me in one case. The usual place for making the puncture is close to the cervix in Douglass' pouch or to one side. If to the side you should locate the uterine artery and puncture just below it. Keep close to the cervix, it is better to wound a uterine vessel than a ureter. You are now prepared to make the puncture. With the index finger at the place of election in the vagina, the middle finger in the rectum to protect it, a stout tenaculum grasping the cervix and held by an assistant, the tumor steadied from above by an assistant pressing on the abdomen forcing it downwards. You will now take a long curved trocar and carry it along the palmer surface of the index finger, and thrust it into the tumor in the direction of the axis of the pelvis. Resistance will cease as soon as you enter the cavity, withdraw the point to see what you have entered; pus immediately begins to flow, replace the point at once to check the flow, which will relax the sac and make further manipulation troublesome. To open the sac and make a free opening for drainage, take a pair of curved sharp pointed scissors, carry them along the palmar surface of the same finger between it and the trocar which still remains in place. Enter them into the sac just beneath and in close proximity with the trocar. As soon as they have entered the cavity remove the trocar. Open the handle of the scissors to enlarge the opening, meanwhile a free flow of pus is escaping. The cavity should now be irrigated. The opening, if necessary, should be enlarged with a pair of uterine dilators. The finger should now be introduced, the cavity explored and any other collections of pus located and emptied through the same incision. If the opposite side is similarly affected it should be treated in the same manner. The cavity should again be irrigated, the water allowed to escape and the cavity loosely packed with iodiform gauze. The vagina should also have a loose packing of gauze after some iodoform and boracic acid powder have been sprinkled in it. A pad of iodoform gauze and piece of cotton should be placed over the vulva and held in place by a T bandage. The vaginal packing should be removed every morning. The packing in the abscess cavity should remain for three days if there are no indications for its removal earlier. The gauze should then be removed each day and the cavity cleansed with peroxide. The incision in the vagina should be kept open until the cavity above has healed. After the cavity has healed there remains in some cases a troublesome endo--metritis and general sub-acute inflammation of the pelvic organs with possibly some thick deposits of lymph or indurations. For the endometritis nothing is so valuable as an aseptic curetting with prohibition from the marital couch for sixty days. For the sub-acute inflammation of the pelvic organs ichthyol and boroglyceride on a wool tampon every or every other night. Internally the perchloride of mercury and iodide of potash alternated with codliver oil and iron. For the deposits negative galvanic electricity is the most potent remedy I have tried. It should be given with a suitable electrode; the negative (pole) electrode introduced into the vagina and the positive on the abdomen. The strength of current should vary, some being able to stand much more than others, ten to thirty milliamperes gradually turned on will be sufficient.

It should be used every second day. If electricity is not available the syrup of the iodide of iron in half teaspoonful doses three times daily will do good, especially if the icthyol and boro-glyceride depleting measures are also used, alternated with painting the cervix and vaginal roof with iodine. The bi-chloride and iodide of potash may be tried.

### TREATMENT BY ABDOMINAL SECTION.

There are three methods applicable to this mode of treatment. Eneucleation, the sac stitched in the abdominal wound, or evacuation per vaginam aided by the hand in the abdomen. I will not burden you with the details of the abdominal surgical technique, as you will have the pleasure of hearing a paper on that subject.

If the abscess sac cannot be clearly defined by a vaginal examination an exploratory incision should be made through the abdominal wall. The abscess should now be carefully studied both by sight and touch in its anatomical relations to surrounding organs and structures. If the patient is in fair condition and the adhesions not very dense, and eneucleation seems possible in a reasonable length of time, this should be done. On the other hand, if the patient seems weak, the adhesions dense, and the pelvis choked with lymph and large abscesses, it is better not to try to eneucleate, but to open per vaginam guided by the hand in the abdomen. The abscess should be treated as when opened per vaginam. If for any reason the sac cannot be eneucleated or brought close enough to the vaginal roof to make it reasonably sure that the pelvic peritoneum will not be extensively soiled with the pus, the sac should be brought in the lower angle of the incision, stitched with chromocised catgut, and the abscess opened, cleansed and packed with iodoform gauze. The after treatment will be the same as when opened per vaginam.

